# 

**Published:** 1874-09-01

**Authors:** 


### BOOK NOTICE.

ARCHIVES OF DERMATOLOGY.—G. P. Putnam & Sons announce a new quarterly journal under this title, to be edited by L. Duncan Buckley, M. D., assisted by a strong corps of collaborators in the various special departments. The first number is to appear about October 1st. Subscription price, $3.00 per annum.

---

### FOR SALE.

My residence and practice — the latter being worth about $3,000 per year. This is a rare chance for a first-class physician.

For particulars, call on or address

L. D. DUNN, M. D.,
Tishkilwa, Illinois.

